# Increased Birth Weight Associated with Regular Pre-Pregnancy Deworming and Weekly Iron-Folic Acid Supplementation for Vietnamese Women

**DOI:** 10.1371/journal.pntd.0001608

**Published:** 2012-04-03

**Authors:** Luca Passerini, Gerard J. Casey, Beverley A. Biggs, Dai T. Cong, Luong B. Phu, Tran Q. Phuc, Marco Carone, Antonio Montresor

**Affiliations:** 1 World Health Organization, Hanoi, Vietnam; 2 Department of Medicine (RMH/WH), The University of Melbourne, The Royal Melbourne Hospital, Parkville, Victoria, Australia; 3 Centre of Clinical Research Excellence in Infectious Diseases (CCREID), The Royal Melbourne Hospital, Parkville, Victoria, Australia; 4 Yen Bai Institute of Malariology, Parasitology and Entomology, Yen Bai Medical College, Yen Bai, Vietnam; 5 National Institute of Malariology, Parasitology and Entomology, Hanoi, Vietnam; 6 Department of Biostatistics, Johns Hopkins Bloomberg School of Public Health, Baltimore, Maryland, United States of America; 7 Department of Control of Neglected Tropical Diseases, World Health Organization, Geneva, Switzerland; George Washington University, United States of America

## Abstract

**Background:**

Hookworm infections are significant public health issues in South-East Asia. In women of reproductive age, chronic hookworm infections cause iron deficiency anaemia, which, upon pregnancy, can lead to intrauterine growth restriction and low birth weight. Low birth weight is an important risk factor for neonatal and infant mortality and morbidity.

**Methodology:**

We investigated the association between neonatal birth weight and a 4-monthly deworming and weekly iron-folic acid supplementation program given to women of reproductive age in north-west Vietnam. The program was made available to all women of reproductive age (estimated 51,623) in two districts in Yen Bai Province for 20 months prior to commencement of birth weight data collection. Data were obtained for births at the district hospitals of the two intervention districts as well as from two control districts where women did not have access to the intervention, but had similar maternal and child health indicators and socio-economic backgrounds. The primary outcome was low birth weight.

**Principal Findings:**

The birth weights of 463 infants born in district hospitals in the intervention (168) and control districts (295) were recorded. Twenty-six months after the program was started, the prevalence of low birth weight was 3% in intervention districts compared to 7.4% in control districts (adjusted odds ratio 0.29, 95% confidence interval 0.10 to 0.81, p = 0.017). The mean birth weight was 124 g (CI 68 - 255 g, p<0.001) greater in the intervention districts compared to control districts.

**Conclusions/Significance:**

The findings of this study suggest that providing women with regular deworming and weekly iron-folic acid supplements before pregnancy is associated with a reduced prevalence of low birth weight in rural Vietnam. The impact of this health system-integrated intervention on birth outcomes should be further evaluated through a more extensive randomised-controlled trial.

## Author Summary

Low birth weight is an important risk factor for neonatal and infant morbidity and mortality and may impact on growth and development. Maternal iron deficiency anaemia contributes to intrauterine growth restriction and low birth weight. Hookworm infections and an iron-depleted diet may lead to iron deficiency anaemia, and both are common in many developing countries. A pilot program of deworming and weekly iron-folic acid supplementation for non-pregnant women aiming to prevent iron deficiency was implemented in northern Vietnam. We compared the birth weight of babies born to women who had had access to the intervention to babies born in districts where the intervention had not been implemented. The mean birth weight of the intervention districts' babies was 124 g more than the control districts' babies; the prevalence of low birth weight was also reduced. These results suggest that providing women with deworming and weekly iron-folic acid supplements before pregnancy is associated with increased birth weight in rural Vietnam. This intervention was provided as a health system integrated program which could be replicated in other at-risk rural areas. If so it could increase the impact of prenatal and antenatal programs, improving the health of both women and newborns.

## Introduction

Low birth weight is widely recognised as a risk factor for neonatal mortality and morbidity, as well as reduced cognitive function and the development of chronic diseases in later life [1]–[3]. Iron deficiency anaemia during pregnancy is an important cause of restricted foetal growth leading to low birth weight and preterm delivery, and also maternal illness and death [4], [5].

It is estimated that more than one third of women in the world are anaemic [6], and iron deficiency is the most common cause of anaemia in the majority of settings [7]. In addition, many of these women live in rural communities of developing countries where intestinal parasitic infections are endemic. Hookworm infections contribute to anaemia severity and persistence by causing chronic blood loss [8], [9]. It has been hypothesized that the anti-helminthic drugs mebendazole and albendazole may have a positive impact on birth outcomes if administered during pregnancy, but conclusive evidence is still lacking. Preventive chemotherapy through mass deworming is recommended when the prevalence of infection with any soil-transmitted helminth exceeds 20% [10]. However, very few countries have promoted routine anti-helminthic treatment in women of reproductive age [11].

Previous studies have reported benefits to maternal and infant health through antenatal supplementation with iron-folic acid supplements and multiple micronutrients [12]–[14], although poor compliance and variable supply have limited the impact of this approach [15]. Preventative weekly iron-folic acid supplementation for women of reproductive age given before pregnancy is effective in improving iron stores and women are less likely to develop iron deficiency anaemia during pregnancy if iron stores are replete at the time of conception [16]. The WHO has recently recommended that weekly iron-folic acid supplementation be made available for women of reproductive age in areas where the prevalence of anaemia in women of reproductive age is above 20% [17].

In Vietnam, the prevalence of anaemia has previously been reportedly as high as 65% in pregnant and 54% in non-pregnant women, and in 2003 it was estimated that 21.8 million people had hookworm infections [18]–[20]. In November 2005 we conducted a survey of anaemia, iron deficiency and hookworm infection in women of reproductive age in Yen Bai province, northern Vietnam. The results showed prevalences to be 38%, 23% and 78% respectively [21]. Anaemia was associated with iron deficiency and meat consumption, however there was no association between hookworm infection and either anaemia or iron deficiency [21]. In response, a pilot weekly iron-folic acid supplementation and deworming program for women of reproductive age was introduced in two districts in May 2006. By September 2007 impact and compliance surveys identified that: 90% of women were taking the weekly iron-folic acid supplements; mean haemoglobin had risen from 122 g/L to 131 g/L; mean serum ferritin levels had risen from 28.1 µg/L to 44.7 µg/L; anaemia, iron deficiency and hookworm infection prevalence had dropped to 20%, 6% and 26% respectively [22], [23].

We hypothesized that the consequent improvement in women's nutritional status would translate to improvements in birth weight of their babies compared to babies born to women in adjacent districts where the intervention was not available.

## Methods

### Ethics Statement

The project was approved by the Human Research Ethics Committee of the National Institute of Malariology, Parasitology and Entomology (Hanoi, Vietnam), the Walter and Eliza Hall Institute of Medical Research (Melbourne, Australia, Project No. 03/07) and Melbourne Health (Melbourne, Australia). The birth weight survey was locally approved by the Yen Bai Ministry of Health. Extensive consultation was undertaken between the project team and community leaders, as well as liaison with village, district and provincial health staff. Village health workers provided participants with information regarding the intervention and signed informed consent was documented. For the birth weight survey, the mother's informed consent was obtained verbally before data collection, as approved by the Yen Bai Ministry of Health Research Committee and in accordance with the Vietnamese Ministry of Health protocols for surveys. Oral consent was documented by the presence of a witness.

### Setting

The pilot intervention was conducted in Tran Yen and Yen Binh districts, with Luc Yen and Nghia Lo as control districts, all in Yen Bai province, a remote, mountainous province in Vietnam with low population density (104 people/km^2^) [24] and poor road and transport infrastructure. Intervention districts were selected based on advice from provincial authorities that they were representative of most other districts in the province in terms of population density and socioeconomic factors. All non-pregnant women between 16 and 45 years in the two districts (estimated 51,623) were targeted. Pregnant women were identified by asking women whether they were pregnant and the timing of their last menstrual period, and were not given deworming treatment if they were or thought they may be pregnant [25]. This protocol was approved by the provincial health authorities. In May 2006 the distribution of iron (FeSO_4_)/folic acid (60/0.4 mg) and 4-monthly albendazole (400 mg) to women was introduced. A detailed account of the protocol has been previously reported [25]. Briefly, iron-folic acid supplements were distributed to village health workers monthly (4 tablets, i.e. weekly consumption for one month) through the administrative strata of the Department of Preventive Medicine. The village health workers then distributed the supplements to individual women either through organized community meetings or home delivery. Provincial authorities chose to distribute 4-monthly deworming treatment through the commune health centres as they felt that this level of the health system was more appropriate for a mass chemotherapy intervention. The intervention was preceded by training of local health workers and delivered with educational activities, distribution of promotional materials to women and community educational meetings [25]. The intervention was not introduced or publicised in other districts of Yen Bai prior to the completion of data collection for this study.

### Study Design

#### Study districts and sample selection

Babies born at district hospitals in the intervention districts of Tran Yen and Yen Binh were selected for the intervention arm. After consultation with the local health authorities, district hospitals in the neighbouring districts of Luc Yen and Nghia Lo were selected for collection of control data on birth outcomes, based on similarity of child and maternal health indicators, as well as socio-economic and demographic statistics. The study sample consisted of all babies born in each district hospital during the period January to June 2008, which constitute about one third of all routine births. The supplementation and deworming program had been operating in the intervention districts for 20 months prior to birth weight data collection. Control districts received routine health services only. Exclusion criteria included complications during pregnancy or labour, twin births or other non-standard pregnancies. Enrolment was confined to births in the district hospitals as it was not logistically possible to monitor all commune health station and home births in the four districts. Women from the districts who gave birth at the provincial hospital were often referred there due to potential complications during delivery and they were also excluded from the survey.

#### Birth weight

Birth weight was measured by midwives, who were specifically trained for the study, using new digital scales (LAICA BF2051). The digital scales were checked before each measurement against standard weights, and regularly calibrated. Weight was measured within 1 hour after birth to the nearest 10 grams; each measurement was performed twice and the mean value reported. Low birth weight was defined as <2500 g [26]. For each delivery, maternity ward staff were asked to complete a questionnaire on birth outcomes and maternal demographic characteristics (socio-economic and educational background, age, pregnancy history).

#### Statistical analysis

All analyses were conducted on an intention-to-treat basis. Multiple gestations, still born babies and those with malformations did not differ between trial and intervention districts (2 vs 1, 1 vs 1 and 1 vs 2 respectively) and were excluded from the analysis. Socio-economic background and baseline characteristics of pregnant women were compared across the study and control group to assess comparability.

The primary outcome was low birth weight. The secondary outcome was mean birth weight. A crude sample size calculation performed prior to initiation of the study indicated that approximately 240 individuals would be required in each group in order to detect, with 80% power, a significant difference in % low birth weight at significance level 0.95 and assuming that 2% and 8% of the pregnancies in the treated and control groups, respectively, would exhibit low birth weight. Odds ratios for low birth weight were estimated using a multiple logistic regression model, adjusting for maternal characteristics and socioeconomic and educational background. Mean birth weight differences were compared using a linear regression model. All models were implemented via generalized estimated equations methodology to account for the correlation due to within-district clustering [27] and robust variance estimators were utilized. Unless otherwise stated, only confounder-adjusted estimates are presented. A total of 9 observations did not include a birth weight measurement (1/169 = 0.6% in treatment group, 8/303 = 2.6% in control group), while a further 47 observations were missing either one or more of the potential confounders included in multivariable analyses (11/168 = 5.9% in control group, 37/295 = 12.5% in treatment group). Missing values were assumed to be missing completely at random and corresponding observations were excluded from analyses. All tests of hypothesis were conducted at confidence level 0.95 with two-sided alternatives. Confidence intervals were constructed by inverting one-sample two-sided z tests. A confidence interval for the number needed to treat was constructed by exponentiating a confidence interval for the logarithm of the number needed to treat; the latter was obtained by resorting to the asymptotic normality of our prevalence estimators as well as the multivariate delta method. Statistical computations were performed using R version 2.8.1.

#### Role of the funding source

Atlantic Philanthropy Inc. sponsored the study and had no role in the study design, data collection, data analysis, data interpretation, or writing of the report.

## Results

Four hundred sixty-three infants were born in the 4 district hospitals during the period of observation (January 2008–Jun 2008), 168 in intervention districts (47.5% females) and 295 (47.2% females) in control districts.

Demographic data for the participants by study arm including maternal age at delivery, parity and socio-economic and educational background are shown in table 1: socio-economic background was similar in the two groups, while women in the intervention districts had higher maternal age but lower level of education.

**Table 1:** Demographic data of study participants.

Indicator	Intervention districts	Control districts	p – value
	(n = 168)^a^^,^ b	(n = 295)^a^^,^ b	
Mean maternal age at delivery	25.8 (4.8)	26.5 (5.4)	<0.001
Median parity	2	2	1
Maternal education			<0.001
*None*	1% (2)	2% (5)	
*Primary*	11% (19)	10% (28)	
*Secondary*	85% (142)	64% (186)	
*Post-Secondary*	3% (5)	24% (68)	
Household socio-economic indicators			0.443
*Owns neither TV nor motorbike*	6% (10)	10% (29)	
*Owns TV only*	13% (22)	15% (46)	
*Owns motorbike only*	3% (5)	4% (11)	
*Owns both*	78% (129)	71% (209)	
Husband's job			0.290
*Agriculture*	80% (131)	71% (206)	
*Trade*	8% (14)	8% (24)	
*Office/teaching*	12% (20)	21% (60)	

a Data are mean (SD), median or % (n).

b Not all women answered all demographic questions.

The prevalence of low birth weight was 3% and 7.4% in the intervention and control districts respectively (Table 2). This equates to a 4.4% absolute and 59% relative lower prevalence of low birth weight (number needed to treat = 23, 8.84–59.11, i.e. one less low birth weight infant for every 23 delivering mothers who had access to deworming and iron-folic acid supplementation prior to pregnancy). The odds ratio of low birth weight comparing infants born to mothers from intervention versus control areas, controlling for the effect of potential confounders and incorporating the possible clustering of inhabitants of a district, was 0.29 (95% CI 0.10 to 0.81, p = 0.017; clustering-adjusted and covariate-unadjusted p = 0.040; clustering-unadjusted and covariate-unadjusted p = 0.077).

**Table 2:** Prevalence and odds ratio of low birth weight; difference in mean birth weight.

Birth Outcome	Intervention districts^a^	Control districts^a^	Odds ratio or difference (95% CI)^b^	p - value
	(n = 168)	(n = 295)		
Low birth weight	3.0% (5)	7.4% (22)	0.29 (0.10–0.81)	0.017
Mean birth weight^c^	3135 (3114–3156)	3011 (2941–3081)	124 (68–255)	<0.001

a Data are % (n) or mean (CI). Mean birth weight is expressed in g.

b Multiple logistic regression analysis for the relative odds of low birth weight was adjusted for maternal age, parity, socio-economic and educational background, as well as clustering within geographical districts.

c Mean birth weights were computed marginally with adjustment for clustering only.

Mean birth weight was 124 g higher in intervention districts (3135 vs 3011 g, CI for mean birth weight increase 68 to 255, p<0.001) (Table 2). Mean birth weight positively correlated with socio-economic background, level of education, maternal age and parity (data not shown).

## Discussion

We present here results that show that the prevalence of low birth weight in infants born to mothers in rural Vietnam who had access to a pre-pregnancy program of 4-monthly albendazole treatment and weekly iron-folic acid was 3.0% compared to 7.4% for those born in neighbouring districts where women did not have access to this intervention. The latter is comparable to the Vietnam national average for low birth weight of 7.0% [28]. In addition, we observed a significantly higher mean birth weight in the intervention group.

While there are numerous studies assessing the benefits of ante-natal iron supplementation for pregnant women, birth outcome and infant health [14], [29], [30], there are few reported long-term studies of the impact of pre-pregnancy weekly iron-folic acid and deworming programs for women of reproductive age on birth outcomes and infant health [31]. Berger et al (2005) reported a prevalence of 2.9% low birth weight infants in a group of women who were participants in a pre-pregnancy weekly iron-folic acid supplementation program who subsequently became pregnant. The prevalence of low birth weight in the daily supplementation arm was 9.3%, however there was no standard treatment control group in the study [32].

Our study has limitations. The study design is not that of a randomized controlled trial, which would allow a more conclusive interpretation of the results. We have tried to adjust for potential confounders but other variables may exist that would bias the results. However, our study was conducted during the implementation of a large scale anaemia and iron deficiency prevention program, and therefore provides information about the impact of the intervention when implemented under field conditions through routine health services.

We are not sure about the exact supplements women took during pregnancy and with what frequency. When questioned, women were unsure of the number and source of free iron-folic acid supplements provided by health workers during pregnancy. Those who bought antenatal nutrition supplements privately did so on the recommendation of a doctor or relation/friend and again were unable to clearly state what the supplements were or how much iron they contained. We therefore cannot exclude a possible bias in the results due to differences in antenatal iron supplementation patterns between the intervention and control districts.

The intervention in Tran Yen/Yen Binh did not have separate arms for iron-folic acid or deworming alone so the relative contribution of each cannot be ascertained. A previous pregnancy supplementation and deworming study suggested that haematinics and anthelminthics had an additive effect on stabilizing haemoglobin during pregnancy when given to 125 Sierra Leone women, with haematinics having the greater effect [33].We find it plausible in our case that iron-folic acid and deworming acted additively or even synergistically, targeting the problem of maternal anaemia at different levels [6]. Regular hookworm control is likely to have complemented iron-folic acid supplementation by reducing iron loss due to chronic hookworm infection. Moreover, the pre-pregnancy population-based approach has been previously shown to result in a gradual and stable improvement in iron status prior to conception [22]. Another significant advantage of regular deworming for non-pregnant women in Vietnam is that Ministry of Health regulations proscribe administering deworming medication during pregnancy.

Another limitation was that we were not able to sample the entire pool of deliveries of mothers from intervention and control districts, as the cost and logistics in such a remote area were beyond the resources available to this study. District hospitals collect about a third of routine deliveries; although this is not a fully comprehensive sample, we believe it is representative enough across the study districts. Furthermore, if selection bias did exist, we assume it would be similar in intervention vs control districts.

It is also important to acknowledge that this intervention would increase the workload of community health workers, which is currently a topic of debate in the international development community. This may challenge the long term sustainability of the implementation and needs to be taken into consideration by health authorities in planning for the intervention.

The data presented here is the result of one of few studies of the impact of pre-pregnancy iron-folic acid supplementation and deworming for non-pregnant women on infant birth weight. Whilst it has been recently suggested that there is a need for stronger, more robust data to support long-term intermittent iron-folic acid supplementation in women of reproductive age [34], there is growing evidence that these programs are not only important [35] but also feasible and implementable in resource constrained settings [23], [25], [31].

The results of our study suggest that the pre-pregnancy combination of deworming and weekly iron-folic acid supplementation for women of reproductive age in northern Vietnam is associated with a reduced incidence of low birth weight and higher mean birth weight. Such a program could potentially represent a high-impact and easily implementable intervention to apply in settings with a high prevalence of hookworm infection and anaemia, for the health of both women and newborns.
